# A survey of core outcome set developers

**DOI:** 10.1186/1745-6215-16-S1-O1

**Published:** 2015-05-29

**Authors:** Elizabeth Gargon, Bridget Young, Paula R Williamson

**Affiliations:** 1 Department of Biostatistics, University of Liverpool, Liverpool, L69 3GA, UK; 2 Department of Psychological Sciences, University of Liverpool, Liverpool, L69 3GL, UK

## Background

A systematic review of core outcome sets (COS) identified 250 reports relating to 198 studies [1]. The review showed that a range of methods have been used, in a variety of ways, to develop core outcome sets. Furthermore, we found that of the 178 studies that described the methods they used to determine the core outcome set, 164 (92%) did not provide an explanation regarding their choice of methodology. To our knowledge, there is little guidance about how to conduct or report COS studies and it is currently uncertain which of these methods are the most suitable, feasible and efficient. It is important to investigate COS developers’ choice of approach as this is a new area of research, and in order to formulate guidance in this area we need to try and understand the current situation, including the influences of methodological choices being made.

## Methods

We have used a mixed methods approach, using qualitative methods (semi-structured interviews) and an online web-based survey. This abstract focusses on the web based survey, the content of which has been informed by the first 9 interviews conducted.

## Results

The survey was sent out to 169 COS developers. Interim results showed that 57/169 COS developers had completed the survey (preliminary response rate of 34%). We asked COS developers to tell us how their COS studies came about. The most frequent responses where because of differences in what is being measured, but also how outcomes were being measured, in trials and research. Methodological decisions were based most commonly on literature (previous work), expert advice or own experience with methods. Challenges of this work included resources (time, funding and technology), achieving consensus, a lack of data and challenges with involving patients in the process.

## Conclusion

This study will contribute to a larger research project that is aiming to develop methodological guidance for COS development. In order to develop this guidance we need to try to understand what factors have informed the ways in which researchers have developed COS. This is the first insight into COS developers choice of methodology and their experiences of the process. These survey results will contribute to a more comprehensive account of COS development, ultimately facilitating the formulation of guidance in this area.
